# Nrf2 null enhances UVB-induced skin inflammation and extracellular matrix damages

**DOI:** 10.1186/2045-3701-4-39

**Published:** 2014-08-05

**Authors:** Constance Lay Lay Saw, Anne Yuqing Yang, Mou-Tuan Huang, Yue Liu, Jong Hun Lee, Tin Oo Khor, Zheng-Yuan Su, Limin Shu, Yaoping Lu, Allan H Conney, Ah-Ng Tony Kong

**Affiliations:** 1 Center for Cancer Prevention Research, Ernest Mario School of Pharmacy, Rutgers, The State University of New Jersey, Piscataway, NJ 08854, USA; 2 Department of Pharmaceutics, Ernest Mario School of Pharmacy, Rutgers, The State University of New Jersey, Piscataway, NJ 08854, USA; 3 Susan Lehman Cullman Laboratory for Cancer Research, Department of Chemical Biology, Ernest Mario School of Pharmacy, Rutgers, The State University of New Jersey, Piscataway, NJ 08854, USA

**Keywords:** Nuclear factor (erythroid-derived 2)-like 2 (NFE2L2 or Nrf2), Ultraviolet, Inflammation, p53, pro-MMP-9, MIP-2, ECM

## Abstract

Nrf2 plays a critical role in defending against oxidative stress and inflammation. We previously reported that Nrf2 confers protection against ultraviolet-B (UVB)-induced inflammation, sunburn reaction, and is involved in sulforaphane-mediated photo-protective effects in the skin. In this study, we aimed to demonstrate the protective role of Nrf2 against inflammation-mediated extracellular matrix (ECM) damage induced by UVB irradiation. Ear biopsy weights were significantly increased in both Nrf2 wild-type (Nrf2 WT) and knockout (Nrf2 KO) mice one week after UVB irradiation. However, these weights increased more significantly in KO mice compared to WT mice, suggesting a greater inflammatory response in KO mice. In addition, we analyzed the protein expression of numerous markers, including macrophage inflammatory protein-2 (MIP-2), pro-matrix metalloproteinase-9 (MMP-9), and p53. p53, a regulator of DNA repair, was overexpressed in Nrf2 KO mice, indicating that the absence of Nrf2 led to more sustained DNA damage. There was also more substantial ECM degradation and increased inflammation in UVB-irradiated Nrf2 KO mice compared to UVB-irradiated WT mice. Furthermore, the protective effects of Nrf2 in response to UVB irradiation were mediated by increased HO-1 protein expression. Collectively, our results show that Nrf2 plays a key role in protecting against UVB irradiation and that the photo-protective effect of Nrf2 is closely related to the inhibition of ECM degradation and inflammation.

## Introduction

The incidence of non-melanoma skin cancer has steadily increased, and ultraviolet (UV) light is one of the major causes of non-melanoma skin cancer. In fact, UV-induced non-melanoma skin cancer is the most common type of cancer in the US and worldwide [1,2]. UVB is the most potent form of UV radiation; exposure to UVB radiation both burns the skin and drives the initiation, promotion, and progression of skin carcinogenesis [3]. UV irradiation induces the delayed expression of UV-responsive genes such as matrix metalloproteinases (MMPs), which degrade macromolecular components of the extracellular matrix (ECM), and these changes represent a hallmark of carcinogenesis and aging [4]. Moreover, altered ECM metabolism has been implicated in various diseases [5]. Oxidative stress, such as that caused by excessive reactive oxygen species (ROS), is caused by UVB damage to DNA, protein, and lipids and can lead to inflammation, gene mutation, and immunosuppression [6,7]. Due to the critical role of ROS in UVB-induced photo-carcinogenesis, ROS detoxification mechanisms have emerged as effective approaches for cancer prevention [8]. Nuclear factor erythroid-2 (NF-E2)-related factor 2 (Nrf2), a member of the cap ‘n’ collar family of redox-sensitive bZIP (basic leucine zipper) proteins, is the major transcriptional regulator of the expression of genes encoding phase II detoxifying/antioxidant enzymes, including heme oxygenase-1 (HO-1) [7]. HO-1 exhibits broad cytoprotective effects in various inflammatory diseases [9] and was shown to play a critical protective role in limiting oxidative damage in a UVB-induced model [10]. MMP-9 was reported to be upregulated in angiogenic dysplasias and invasive cancers of the epidermis in a mouse model of multistage tumorigenesis. Notably, MMP-9 is predominantly expressed in neutrophils, macrophages, and mast cells rather than oncogene-positive neoplastic cells [11]. It has also been reported that the disruption of Nrf2 enhanced the up-regulation of key inflammatory transcription factors, such as nuclear factor kappa B (NF-κB) and MMP-9 [12], and increased expression of macrophage inflammatory protein-2 (MIP-2), a neutrophil chemotactic factor, was observed in response to 12-*O*-tetradecanoylphorbol-13-acetate (TPA)-induced epidermal hyperplasia in mice [13]. The p53 tumor suppressor gene plays an important role in protecting cells against DNA damage and strand breaks [14], and the impact of UVB on p53 has been experimentally verified [15]. Moreover, p53 is known to be activated by DNA damage [14], oxidative stress [16], and inflammation [17,18].

We previously reported the critical role of nuclear Nrf2 in the classical 2-stage model of skin carcinogenesis induced by the chemical carcinogen 7,12-dimethylbenz(a)anthracene (DMBA) and the tumor promoter TPA in Nrf2 knockout (Nrf2 KO) and wild-type (WT) mice [19]. We also recently reported that Nrf2 plays an important role in the SFN-mediated protective effects against UVB-induced inflammation [20]. To explore the potential impact of Nrf2 on UVB-induced inflammatory ECM damage in the skin, we studied the potential biomarkers involved in ECM degradation and inflammation in Nrf2 KO and WT mice, as these markers may be relevant to human skin carcinogenesis and photoaging [8,21-24]. Based on this context, our current study aimed to ascertain the role of Nrf2 in UVB-induced ECM damage by focusing on inflammation and DNA repair in Nrf2 KO and Nrf2 WT mice.

## Materials and methods

### Animals

Male and female Nrf2 KO and WT C57BL/6 mice were obtained as previously described [19]. The offspring of the eighth generation of Nrf2 KO mice on the C57BL/6 background were used in this study. The genotype of each animal was confirmed using DNA extracted from the tail and analyzed by PCR. Nrf2 WT mice were purchased from The Jackson Laboratory (Bar Harbor, ME, USA). Mice were housed at the Rutgers Animal Facility, maintained under 12-h light/dark cycles, and provided *ad libitum* access to food and water. Animal experiments were performed in accordance with the Guide for the Care and Use of Laboratory Animals. All animal experimental procedures were approved by the Animal Care and Use Committee of Rutgers, The State University of New Jersey (Protocol Number: 04-003).

### UV lamps

To induce skin inflammation, animals received a single dose of UVB light (300 mJ/cm^2^) as previously described [20,25]. These UV lamps (FS72T12-UVB-HO; National Biological Corp., Twinsburg, OH, USA) emit little or no radiation < 280 nm and > 375 nm. The lamps emit UVB (280-320 nm; 75-80% of total energy) and UVA (320-375 nm; 20-25% of total energy), as described in our previous studies [20,25]. The dose of UVB was quantified using a UVB Spectra 305 dosimeter (Daavlin Co., Bryan, OH, USA). The radiation was calibrated with an IL-1700 research radiometer/photometer (International Light Inc., Newburyport, MA, USA).

### Experimental design

Experiments were performed in Nrf2 WT and KO mice to determine whether a single dose of 300 mJ/cm^2^ UVB irradiation would be sufficient to induce inflammatory reactions in the skin. Seven days after UVB irradiation, ear punches (6 mm in diameter) were obtained and weighed. Two sets of experiments were conducted in Nrf2 WT and KO mice after UVB exposure (at 8 h and 8 days). For each experiment, 4 groups of mice were evaluated: (1) no UVB in Nrf2 WT mice; (2) UVB in Nrf2 WT mice; (3) no UVB in Nrf2 KO mice; and (4) UVB in Nrf2 KO mice. A minimum of three animals was included in each group. The hair on the dorsal region of each mouse was removed 2 days before UVB irradiation, as previously described [20]. At the end of the experiment, the mice were sacrificed by cervical dislocation, and the skin samples were frozen in liquid nitrogen and stored at -80°C.

### Preparation of skin specimens and histological examination

Skin samples were obtained from the dorsal area of the mouse and were placed in 10% phosphate-buffered formalin at room temperature overnight. The samples were then dehydrated in increasing concentrations (80, 95, and 100%) of ethanol, cleared in xylene, and embedded in Paraplast Plus (Fisher Scientific, Pittsburgh, PA, USA). Four-micrometer serial sections were cut from the skin block, deparaffinized, rehydrated, and stained with hematoxylin and eosin (H&E). The H&E sections were examined under a light microscope (Nikon Eclipse E600, Japan).

### ELISA for pro-inflammatory proteins and p53

The protein levels of pro-inflammatory cytokines and p53 were determined using two-site sandwich enzyme-linked immunosorbent assays (ELISAs). Dorsal skin tissues were homogenized in phosphate-buffered saline (PBS) containing 0.4 M NaCl, 0.05% Tween-20, 0.5% bovine serum albumin, 0.1 mM phenylmethylsulphonyl fluoride, 0.1 mM benzethonium chloride, 10 mM EDTA, and 200 KIU aprotinin per mL. The homogenates were centrifuged at 12,000 × *g* for 15 min at 4°C. The supernatant was used for the determination of cytokines levels. Pro-matrix metalloproteinase-9 (pro-MMP-9; DY909), macrophage inflammatory protein-2 (MIP-2; DY452), and p53 (DYC1746-2) kits were purchased from R&D Systems, Inc. (Minneapolis, MN, USA).

### Western blotting

The protein samples were prepared as described above for the ELISAs. The protein concentration was measured using Bio-Rad protein assay reagent (Hercules, CA, USA). Equal amounts of protein (50 μg) from each sample were loaded onto a homemade 10% Tris/glycine SDS-polyacrylamide gel containing 0.1% SDS and run at 200 V for 1 h. The proteins were transferred to a nitrocellulose membrane by electroblotting using a Bio-Rad semidry transfer blotting apparatus over 1.5 h at 90 V (Bio-Rad, Hercules, CA, USA). The membrane was blocked with blocking buffer (Odyssey system, LI-COR Biosciences, Lincoln, NE, USA) for 1 h at room temperature. The primary antibodies were diluted in the same blocking buffer and were added to detect the target proteins. After an overnight incubation at 4°C, the membrane was washed with TBST (Tris-buffered saline and 0.05% Tween-20) and then incubated with the secondary antibody. Finally, the target protein bands were visualized with an Odyssey infrared imager. Antibodies against β-actin and HO-1 were purchased from Santa Cruz Biotechnology, Inc. (CA, USA). Densitometry was performed using the Image J software (version 1.44, National Institute of Health, USA). The relative protein expression was obtained by first calculating the relative intensity compared to the control (no UVB treatment) sample on the same blot and then normalizing this value to the intensity of β-actin from the same sample.

### Data presentation and statistical analysis

The data are presented as the mean ± standard error of the mean (SEM), except as otherwise stated. Student’s *t*-test was used to determine statistically significant differences between groups. A p value < 0.05 was considered statistically significant.

## Results

### A single dose of 300 mJ/cm^2^ UVB significantly increased the ear biopsy weight, and Nrf2 KO mice were more susceptible to UVB-induced skin edema

**Figure 1:**
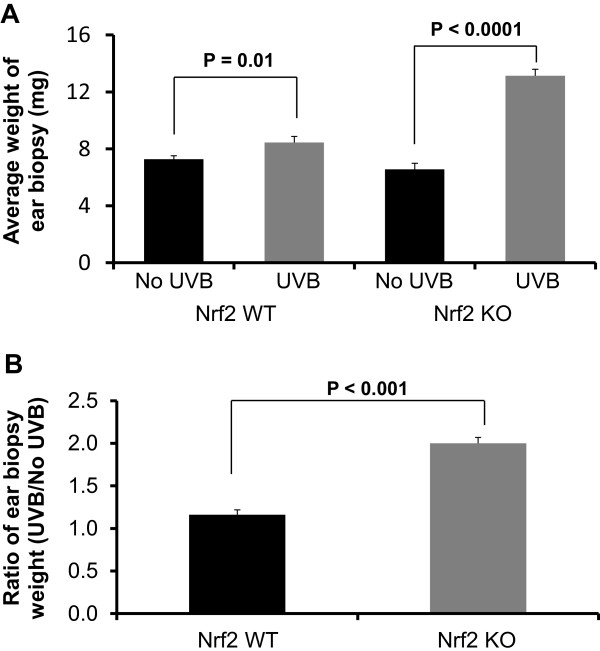
**Ear biopsy weights in Nrf2 WT and KO mice after UVB exposure. (A)** Nrf2WT and KO mice were treated with or without a single dose of UVB (300 mJ/cm^2^). The mice were sacrificed 1 week after UVB irradiation. Ear punches (6 mm in diameter) were taken and weighed. **(B)** The ratio of the ear punch weight in UVB-treated versus control skin suggested that Nrf2 significantly protected against the UVB-induced increase in ear punch weight (p < 0.05). The data are presented as the mean ± SEM.

To determine the effect of Nrf2 on UVB-induced trauma to mouse skin, we irritated mouse skin with a single dose of UVB (300 mJ/cm^2^) and measured the ear biopsy weight 1 week after exposure. In the untreated group, the average ear weights of the Nrf2 WT and KO mice were 7.28 ± 0.24 mg and 6.57 ± 0.42 mg, respectively. In the UVB-treated group, these weights increased to 8.45 ± 0.42 mg in Nrf2 WT mice and to 13.13 ± 0.45 mg in Nrf2 KO mice (Figure 1A). UVB exposure significantly increased the ear biopsy weight in Nrf2 KO mice by nearly 100%, whereas there was only a 16% increase observed in Nrf2 WT mice. The ear biopsy weight ratio indicated that Nrf2 KO mice were more susceptible to UVB-induced skin edema (Figure 1B). These data demonstrated that a single dose of UVB (300 mJ/cm^2^) was sufficient to cause UVB-induced inflammatory damage to mouse skin, especially in Nrf2 KO mice. This single dose of UVB was used in all the following experiments.

### The absence of Nrf2 gene expression increased the UVB-induced skin thickness

**Figure 2:**
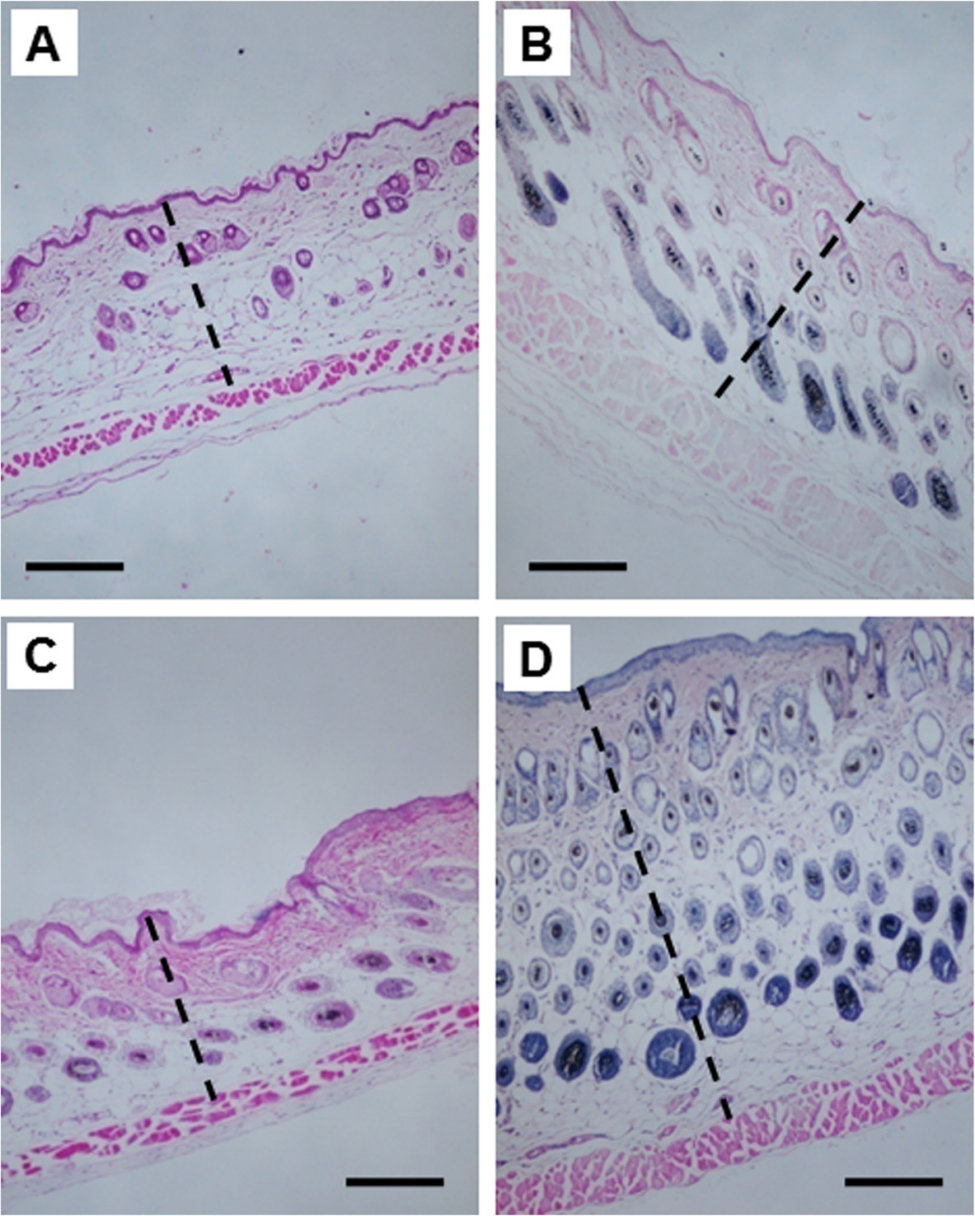
**Skin thickness in Nrf2 WT versus Nrf2 KO mice after UVB exposure.** H&E staining was performed in skin samples 8 days after treatment with a single dose of UVB (300 mJ/cm^2^). WT mice were treated **(A)** without UVB or **(B)** with UVB. KO mice were treated **(C)** without UVB or **(D)** with UVB. The horizontal line represents 200 μm, and the broken lines indicate the skin thickness. Original magnification, ×400. The Nrf2 KO mice exhibited more edema and inflammatory changes, such as increased skin thickness, whereas the Nrf2 WT mice demonstrated fewer changes; these data correlated with the ear punch weights (Figure 1) and other biomarkers (Figures 3, 4, 5).

To determine the effect of Nrf2 in mouse skin exposed to UVB irritation, we treated the dorsal skin area with a single dose of UVB (300 mJ/cm^2^) and took a skin biopsy 8 days later. Nrf2 KO mice exhibited a significant increase in skin thickness 8 days after UVB irradiation (Figure 2), and a single dose of UVB (300 mJ/cm^2^) led to a greater increase in skin thickness in Nrf2 KO mice compared to Nrf2 WT mice (Figure 2).

### Nrf2 KO mice were significantly more susceptible to UVB-induced inflammation and ECM degradation

**Figure 3:**
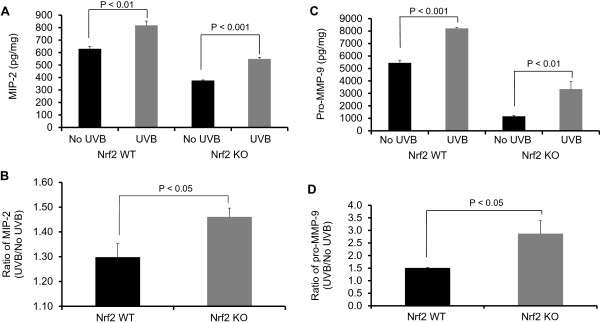
**MIP-2 and Pro-MMP-9 expression in Nrf2 WT versus Nrf2 KO mice after UVB exposure.** Nrf2 WT and KO mice were irradiated with a single dose of UVB (300 mJ/cm^2^). After 8 h, **(A)** the MIP-2 expression and **(B)** the ratios of MIP-2 protein levels in WT and Nrf2 KO mice; **(C)** the Pro-MMP-9 expression and **(D)** the ratios of Pro-MMP-9 protein levels in UVB-treated versus control skin (No UVB-treated) were determined; Nrf2 significantly protected against UVB-induced the MIP-2 and pro-MMP-9 expression (p < 0.05). The data are presented as the mean ± SEM.

To characterize the UVB-induced inflammatory damage to the ECM, we measured the expression of biomarkers related to inflammation (MIP-2) and ECM degradation (pro-MMP-9) 8 h after UVB exposure. A single dose of UVB (300 mJ/cm^2^) significantly upregulated the protein expression of MIP-2 and pro-MMP-9 in both Nrf2 WT and KO mice (Figures 3A and 3C). However, after UVB irradiation, these biomarkers were more significantly upregulated in Nrf2 KO mice compared to WT mice (Figures 3B and 3D).

### Nrf2 KO mice were more susceptible to UVB-induced upregulation of p53

**Figure 4:**
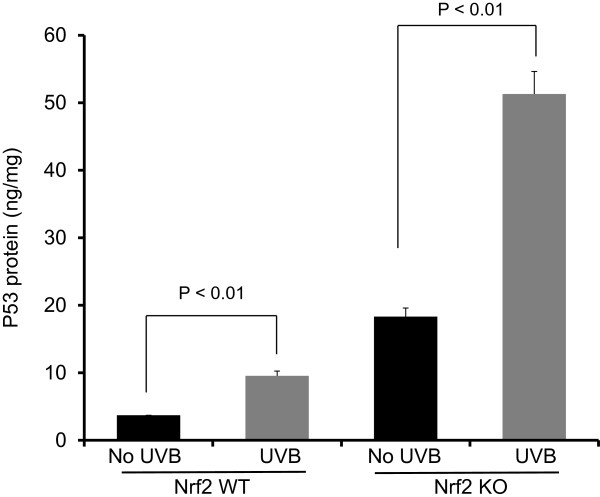
**p53 expression in Nrf2 WT versus KO mice after UVB exposure.** Nrf2 WT and KO mice were irradiated with a single dose of UVB (300 mJ/cm^2^). p53 protein levels increased in WT and KO mice 8 h after UVB irradiation; however, there was a greater increase in p53 expression in Nrf2 KO compared to WT mice. The data are presented as the mean ± SEM.

To evaluate sustained DNA damage, we examined p53 protein expression 8 h after UVB irradiation. In both Nrf2 WT and KO mice, a single dose of UVB (300 mJ/cm^2^) significantly increased p53 protein expression compared to untreated skin (Figure 4). However, there was a greater increase in p53 expression in Nrf2 KO mice compared to Nrf2 WT mice.

### UVB increased expression of the Nrf2 target HO-1, an antioxidant biomarker, in Nrf2 WT mice compared to Nrf2 KO mice

**Figure 5:**
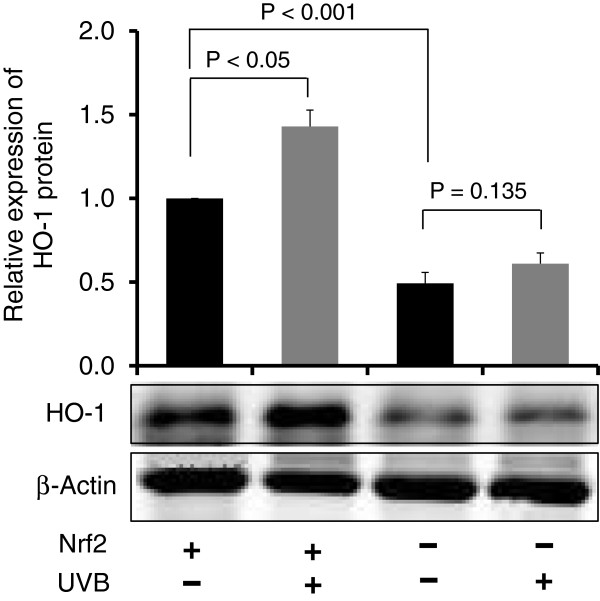
**Nrf2-targeted antioxidant biomarkers in Nrf2 WT versus KO mice after UVB exposure.** Nrf2 WT and KO mice were irradiated with a single dose of UVB (300 mJ/cm^2^). Expression of the Nrf2-regulated antioxidant protein HO-1 was measured in the skin. β-Actin served as the endogenous housekeeping protein. Protein samples (50 μg) were subjected to western blotting, and the relative intensity was calculated by dividing the intensity of the protein band by that of the control sample on the same blot and then normalizing against the intensity of β-actin on the same membrane. After UVB irradiation, HO-1 expression was increased in WT mice but increased in much lesser extent in KO mice.

To elucidate the mechanism responsible for the photo-protective effect of Nrf2 in UVB-exposed mice, Nrf2 WT and KO mice were exposed to UVB irradiation and sacrificed 8 h later. We measured the expression of HO-1, an anti-oxidative enzyme downstream of Nrf2, in Nrf2 WT and KO mice and compared these levels to those observed in untreated control Nrf2 WT and KO mice. Compared to untreated WT mice, HO-1 protein expression was significantly increased in UVB-treated Nrf2 WT mice (p < 0.05), whereas HO-1 expression was not significantly altered in Nrf2 KO mice (Figure 5). In particular, UVB exposure increased HO-1 expression by 43% in Nrf2 WT mice, which was much higher than the 24% increase observed in Nrf2 KO mice.

## Discussion

In the present study, we investigated the photo-protective role of Nrf2 in inflammation-mediated ECM damage induced by UV, and our results indicated that Nrf2 KO mice were more susceptible to UVB radiation. Erythema has long been used as an indicator of UV-induced inflammation [26] and has been characterized as a reliable, reproducible and quantitative measurement of the degree of UV-induced inflammation [27]. Thus, measuring the weight or thickness of irritated skin is a reliable indicator of inflammation [20,28]. The results presented here demonstrated that UVB exposure significantly increased the ear biopsy weight in Nrf2 KO mice by almost 100%, whereas the ear biopsy weight only increased by 16% in Nrf2 WT mice. Moreover, the ratio of ear biopsy weights suggested that Nrf2 KO mice were more susceptible to UVB-induced skin edema (Figure 1). In addition, Nrf2 KO mice exhibited a greater increase in skin thickness after UVB exposure (Figure 2), which suggested that the loss of Nrf2 resulted in increased sensitivity to UVB radiation, thereby implying that there was more active inflammation in Nrf2-deficient mice. Therefore, we performed various biological assays to demonstrate that the observed UVB-induced responses were related to inflammation, ECM stability, and DNA damage. It has been reported in *in vivo* studies of human skin that UV irradiation significantly affects the coordinated regulation of various MMPs; after a single exposure to UV, MMP-9 activity increased 4-fold compared to non-UV irradiated human skin in a time-dependent manner [29]. MIP-2, a key mediator of neutrophil recruitment, is involved in the early infiltration of neutrophils into UVB-exposed skin [30]. In our study, UVB irradiation significantly enhanced the expression of MIP-2 in both Nrf2 WT and KO mice (Figure 3A). However, the increase in MIP-2 expression was much greater in Nrf2 KO mice compared to WT mice (Figure 3B). These MIP-2 findings correlate with those of a previous study in which MIP-2 expression was increased in mice after UVB irradiation [30]. Photoaged skin displays prominent alterations in the collagenous ECM of connective tissue [29], and the detrimental effects of ECM degradation include diminished structural integrity, impaired wound healing, loss of cell viability, and cancer metastasis [31]. There is growing interest in understanding the role of aberrant ECM modifications, especially regarding the “cancer stem cell niche” and the “metastatic niche” during key stages of cancer progression [7,32]. Pro-MMP-9 expression was increased significantly in Nrf2 KO mice compared to WT mice (Figures 3C and 3D), and these results indicated that the absence of Nrf2 augmented the expression of matrix-degrading proteinases, including pro-MMP-9, in response to UVB irradiation. Furthermore, many ECM fragments that are created via degradation by various MMPs act as chemotactic factors that cause endothelial and inflammatory cells to migrate into areas of active tumor cell proliferation and growth [33]. Thus, the photo-protective effect of Nrf2 was mediated by a reduction in MMP-9 expression. The p53 tumor suppressor gene is typically activated by DNA damage [14], oxidative stress [16], and/or inflammation [17,18], and we observed that both of Nrf2 WT and KO mice exhibited increased p53 expression in response to UVB exposure, although the p53 expression was higher in KO mice. Regarding the role of p53 in DNA repair, increased p53 expression correlates with increased DNA damage, suggesting that the loss of Nrf2 may fail to protect DNA but could enable abundant p53 to repair the DNA damage. Indeed, a previous study showed that the extent of DNA damage correlates with the amount of UV exposure in various skin cells and models [34]. In our study, the higher p53 expression observed in Nrf2 KO mice after UVB exposure could be an indicator of more sustained DNA damage in need of repair. HO-1 is a downstream antioxidant enzyme that is regulated by Nrf2 and has a broad cytoprotective effect in various inflammatory diseases and UVB irradiation [9,10]. In our study, UVB exposure enhanced HO-1 expression by 43% in Nrf2 WT mice and by 24% in Nrf2 KO mice (Figure 5), which indicated that the photo-protective effect of Nrf2 was mediated by HO-1.

## Conclusions

UVB irradiation induces aberrant biological responses, such as increased skin erythema and thickness, up-regulated expression of inflammatory and ECM-degrading enzymes, and augmented DNA damage. According to our results, these biological responses were milder in WT mice compared to Nrf2 KO mice. In addition, our UVB-induced mechanistic study revealed that the protective effect of Nrf2 was mediated by HO-1, and these findings suggest that Nrf2 plays a key role in protecting against UVB irradiation. Moreover, our study is the first to demonstrate that the photo-protective effect of Nrf2 is closely related to the inhibition of ECM degradation and inflammation through the overexpression of HO-1.

## Competing interests

The authors declare that they have no competing interests.

## Authors’ contributions

CLLS, MTH and YL conceived the research design and carried out the experiments, as well as analyzed the data. CLLS, AYY, JHL, TOK, ZYS, LS and ANTK wrote, reviewed and/or revised this manuscript. MTH, YPL, AHC and ANTK provided administrative, technical and material support. All authors read and approved the final manuscript.
